# Outcomes of Bone Marrow Compared to Peripheral Blood for Haploidentical Transplantation

**DOI:** 10.3390/jcm10132843

**Published:** 2021-06-27

**Authors:** Nidhi Sharma, Muhammad Salman Faisal, Qiuhong Zhao, Justin Jiang, Patrick Elder, Don M. Benson, Ashley Rosko, Maria Chaudhry, Naresh Bumma, Abdullah Khan, Srinivas Devarakonda, Sumithira Vasu, Samantha Jaglowski, Alice S. Mims, Hannah Choe, Karilyn Larkin, Jonathan E. Brammer, Sarah Wall, Nicole Grieselhuber, Ayman Saad, Sam Penza, Audrey M. Sigmund, Yvonne A. Efebera

**Affiliations:** 1 Division of Hematology, Department of Internal Medicine, The Ohio State University, Columbus, OH 43210, USA; Muhammad.Faisal2@osumc.edu (M.S.F.); Qiuhong.Zhao@osumc.edu (Q.Z.); Patrick.Elder@osumc.edu (P.E.); Don.Benson@osumc.edu (D.M.B.); Ashley.Rosko@osumc.edu (A.R.); Maria.Chaudhry@osumc.edu (M.C.); Naresh.Bumma@osumc.edu (N.B.); Abdullah.Khan@osumc.edu (A.K.); Srinivas.Devarakonda@osumc.edu (S.D.); Sumithira.Vasu@osumc.edu (S.V.); Samantha.Jaglowski@osumc.edu (S.J.); Alice.Mims@osumc.edu (A.S.M.); Hannah.Choe@osumc.edu (H.C.); Karilyn.Larkin@osumc.edu (K.L.); Jonathan.Brammer@osumc.edu (J.E.B.); Sarah.Wall@osumc.edu (S.W.); Nicole.Grieselhuber@osumc.edu (N.G.); Ayman.Saad@osumc.edu (A.S.); Sam.Penza@osumc.edu (S.P.); Audrey.Sigmund@osumc.edu (A.M.S.); Yvonne.Efebera@ohiohealth.com (Y.A.E.); 2 College of Medicine, The Ohio State University, Columbus, OH 43210, USA; Justin.Jiang@osumc.edu

**Keywords:** haploidentical transplantation, peripheral blood, bone marrow, allogenic transplantation

## Abstract

Allogeneic hematopoietic cell transplantation (allo-HCT) from a haploidentical (haplo) donor has emerged as a suitable alternative in the absence of a matched donor. However, haplo-HCT patients have a higher risk of graft-versus-host disease (GVHD). Hence, bone marrow (BM) stem cell source and post-transplant cyclophosphamide (PTCy) have been routinely used to help mitigate this. Due to ease of collection, peripheral blood (PB) stem cells are increasingly being considered for haplo-HCT. We retrospectively analyzed 74 patients (42 BM and 32 PB) who underwent haplo-HCT at Ohio State University from 2009 to 2018. Median age at transplant was 60 years (yrs) for BM and 54 yrs for PB, (*p* = 0.45). There was no difference in OS (*p* = 0.13) and NRM (*p* = 0.75) as well as PFS (*p* = 0.10) or GRFS (*p* = 0.90) between the groups. The BM cohort showed a 3-year OS rate of 63% (95% confidence interval (CI): 46–76), and 3-year PFS of 49% (95% CI: 33–63). For the PB group, 3-year OS and PFS were 78% (95% CI: 59–89) and 68% (95% CI: 49–82), respectively. There were no differences in the incidence of acute GVHD (grade II-IV) (*p* = 0.31) and chronic GVHD (*p* = 0.18). Patients receiving BM had a significantly higher risk for relapse with relapse rates by 2 years at 36% (95% CI: 22–50) vs. 16% (95% CI: 6–31) for PB (*p* = 0.03). The findings from this study suggest that PB is an excellent alternative to BM for haplo-HCT.

## 1. Introduction

Allogeneic hematopoietic stem cell transplant (allo-HCT) is a potentially life-saving therapy for hematologic malignancies and other diseases. An HLA-matched sibling (MRD) is an optimal donor, but only 30% of people have such an available donor [1]. For those patients who do not have an MRD, search for matched unrelated donors (MUD) through donor registries such as the National Marrow Donor Program (NMDP) is the best option. However, the probability of finding an 8/8 MUD (HLA A, B, C, DRB1) varies significantly based on ethnicity, with probability of 75% for Caucasians but only 16–19% for African Americans [2]. Haploidentical (haplo) donor transplant offers a great alternative donor option due to its universal availability. The majority of patients have a viable haplo donor, with 96.6% of patients having at least one haplo donor with an average of 2.5 haplo donors in a John Hopkins University 2006–2011 cohort [3]. The initial protocol developed for haplo-HCT involved profound T-cell depletion with myeloablative conditioning developed by the Perugia group [4]. However, there were significant limitations including long-lasting immunodeficiency resulting in extraordinary non-relapse mortality (NRM) linked to infection and inadequate anti-leukemia effects resulting in early relapsed disease.

One study by Luznik et al. evaluated the outcomes of 68 patients with high-risk hematological malignancies or paroxysmal nocturnal hemoglobinuria (PNH) undergoing haplo transplant with post-transplantation cyclophosphamide (PTCy). Patients received non-myeloablative conditioning followed by a haplo transplant with bone marrow (BM) source. They were given either a single dose of PTCy on day (D)+3 (Seattle *n* = 28) or two doses on D+3 and D+4 (Baltimore, *n* = 40). All patients also received tacrolimus until day +180 and mycophenolate until day +35. The incidence of graft failure was 9/66 (13%). The incidence of acute GVHD (aGVHD) ≥ grade 3 was only 6% by day +200, and the incidence of extensive chronic GVHD (cGVHD) was 5% in patients who had received two doses of PTCy. NRM at one year was 15%. Overall survival (OS) at two years was 36% [5]. Given that BM grafts have been shown to have a lower incidence of cGVHD [6,7], BM was utilized as the graft source both in this study [5] and in the phase 2 BMT-CTN 0603 trial [8] in an effort to mitigate GVHD incidence.

While prior studies have primarily used BM source for haplo transplants, BM harvest is not without risk and requires significant coordination of logistics and resource utilization. Thus, programs are exploring the use of peripheral blood (PB) stem cells as a source in haplo transplants. In an early clinical study using PB stem cell source, the cumulative incidence of aGVHD at one year was 53% for grade II and 8% for grade III [9]. In a multicenter retrospective European Society for Blood and Marrow Transplantation (EBMT) analysis, aGVHD was seen more frequently in patients who received PB than BM, however, no difference was found in OS, relapse rate, or NRM [10]. A Center for International Blood and Marrow Transplant Research (CIBMTR) analysis in 2017 retrospectively reviewed 681 consecutive patients who received BM (*n* = 481) or PB (*n* = 190) with PTCy between 2009 and 2014. The risks of acute and chronic GVHD were lower in the BM group than the PB group (*p* < 0.001 in both), while the relapse rate was higher in the BM group (*p* = 0.009) [11]. A recent meta-analysis of 14 studies, was also performed, which included four comparative retrospective reports and ten single-arm reports, with a total of 1759 patients who received PTCy haplo-HCT (462 patients received PB, 1297 patients received BM). The analysis showed significantly higher incidence of grade III-IV aGVHD (OR = 1.741, 95% CI 1.032–2.938) and II-IV aGVHD (OR = 1.778, 95% CI 1.314, 2.406) in the PB group. No significant differences were found on the incidence of relapse, 2-year OS and disease-free survival (DFS), and cGVHD between PB and BM [12].

There currently are no published prospective studies comparing BM versus PB stem cell source for haplo-HCT, and consequently data from retrospective analyses have helped guide the use of PB stem cell source. We therefore present our data on the effects of stem cell sources on the outcomes of haplo-HCT at our center in patients who received unmanipulated BM or PB stem cells followed by PTCy.

## 2. Methods

The bone marrow transplant registry was queried to establish a list of all patients who received allo-HCT at the Ohio State University Comprehensive Cancer Center between 2009 and 2018. A total of 74 patients were included in the final analysis. A retrospective review was conducted and all data was verified through medical chart review. Patients who received T-cell depleted grafts were excluded from the analysis. All patients received PTCy and an FK inhibitor with mycophenolate for GVHD prophylaxis.

### Statistical Methods

The study’s primary endpoints were OS and progression-free survival (PFS). Other study endpoints included relapse, aGVHD (grade II–IV), and cGVHD, NRM, and GVHD-free relapse-free survival (GRFS). All endpoints were measured from the time of transplant. Patient, disease, and transplant-related characteristics were compared between the two groups (BM versus PB) using the Mann–Whitney U test for continuous variables and chi-squared or Fisher’s exact test for categorical variables. The probabilities of OS, PFS, and GRFS were calculated using the Kaplan–Meier method and compared using the log-rank test. Cumulative incidence rates for relapse, NRM, and acute and chronic GVHD were estimated and compared using Gray’s test, accounting for competing risks. The competing risk for relapse was death; the competing risks for GVHD were relapse and death, while the competing risk for NRM was disease-related deaths. Univariable models were conducted using either the Cox proportional hazards model or Fine and Gray competing risk regression models to estimate the associations between transplant tissues (PB versus BM) and corresponding outcomes. Patient demographical and disease characteristic variables were evaluated for potential confounding effect in the associations between transplant tissues and outcomes. Confounders were included in final multivariable models to further estimate the adjusted effect of PB versus BM over corresponding outcomes. The significance level was set at 0.05 and all *p*-values presented were from two-sided tests. All analyses were conducted using Stata 16 (College Station, TX, USA).

## 3. Results

### 3.1. Patient Characteristics

A total of 74 patients (42 BM and 32 PB) underwent haplo-HCT between 2009 to 2018 and received PTCy. The median age at transplant was 57 years (60 vs. 54 years in BM vs. PB groups, *p* = 0.45). The donor’s median age was 31 years (34 vs. 28 years in BM vs. PB group, *p* = 0.28). Forty-six patients (62%) were male. The majority of donors were male, 74.3% (55/74); (81% (34/42) in the BM group vs. 65.6% (21/32) in the PB group). The most common diagnosis across both groups was acute myeloid leukemia (33.8%). Seventy-four percent of patients received fludarabine/cyclophosphamide/total body irradiation (FluCyTBI) as their conditioning regimen. The other regimens were fludarabine/busulphan/thiotepa (FluBU) (18.9%), cyclophosphamide/total body irradiation (CyTBI) (1.4%), fludarabine/melphalan (FluMel)(1.4%) and fludarabine/total body irradiation (FluTBI) (4.1%). There was no difference in conditioning regimen between the 2 groups. All patients received PTCy with tacrolimus (or sirolimus) and mycophenolate for GVHD prophylaxis.

Baseline characteristics are described in Table 1. As expected, the PB had a higher dose of CD34+ stem cells (median dose 3.7 million cells/kg in the BM graft vs. 9.2 million cells/kg in PB, *p* < 0.001) as well as a higher dose of CD3+ T-cells (0.4 million cells/kg in BM vs. 2.3 million cells/kg in PB, *p* < 0.001).

### 3.2. Post-Transplant Outcomes

The median time to neutrophil engraftment was 17.5 days in the BM group vs. 16 days in the PB group (*p* = 0.09), while the median time to platelet engraftment was 29 days in the BM group vs. 20 days in PB group (*p* < 0.001). Thirty-five patients (83.3%) in the BM group achieved CR after transplant vs. 31 (96.9%) in the PB group (*p* = 0.18). No patients developed veno-occlusive disease (VOD) in either group. The incidences of complications, including pulmonary infection, bacteremia, viral reactivation, and fungemia within 100 days post-transplant were similar in both groups and are detailed in Table 2. There was only one graft failure observed in the PB group.

### 3.3. Survival Outcomes

**Figure 1:**
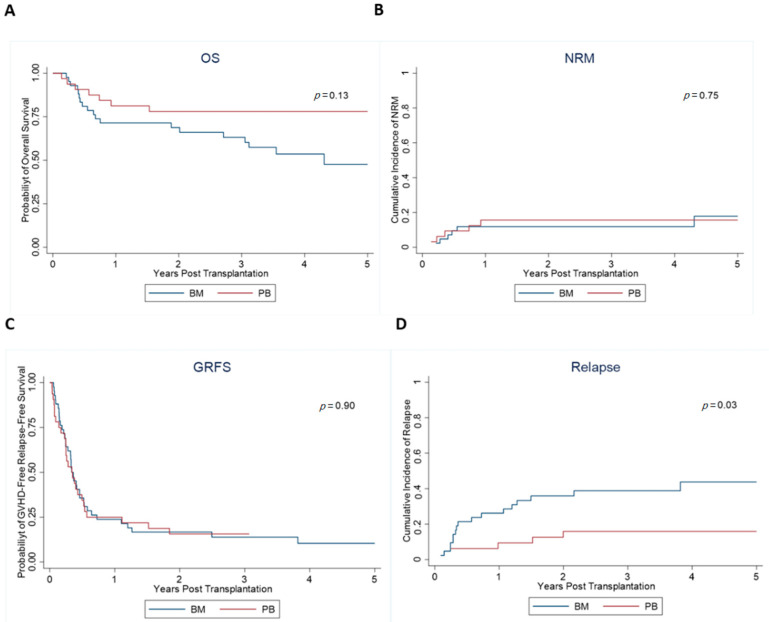
Comparison of survival outcomes between BM and PB grafts. (**A**) probability of overall survival, (**B**) non-relapse mortality, (**C**) GVHD free relapse free survival, and (**D**) cumulative incidence of relapse.

The median follow up in the BM group was 3.6 years, versus 2.9 years in the PB group. There was no difference in OS (*p* = 0.13) and NRM (*p* = 0.75) as well as PFS (*p* = 0.10) or GRFS (*p* = 0.90) between the groups (Figure 1). The median OS of the PB group was not reached (NR). It was 4.3 years in the BM group (95% CI 2.0-NR) (*p* = 0.13). The probability of three year survival was 63% (95% CI 46–76%) in the BM group vs. 78% (95% CI 59–89%) in the PB group (*p* value = 0.16). The median PFS was 2.2 years (95% CI 0.6-NR) in the BM group vs. NR (95% CI 2.0-NR) in the PB group. The probability of 3-year PFS was 49% (95% CI: 33–63%) in the BM group vs. 68% (95% CI 49–82%) in the PB group (*p* value = 0.09). The incidence of NRM at one year was 12% (95% CI 4–24%) in the BM group vs. 16% (95% CI 6–30%) in the PB group (PB vs. BM HR = 1.21 (95% CI: 0.38–3.89, *p* = 0.75). PB graft had a reduced risk of relapse compared to BM graft. The incidence of relapse at one year was 26% (95% CI 14–40%) in the BM group vs. 9% (95% CI 2–22%) in the PB group (PB vs. BM HR = 0.33 (95% CI: 0.13–0.88, *p* = 0.03) (Figure 1). There were 27 deaths in the entire cohort and 4 of 27 were due to infection.

### 3.4. Acute and Chronic GVHD

Grade II-IV aGVHD at day 180 was 57% (95% CI 41–70%) in BM vs. 63% (95% CI 44–77%) in PB, while grade III-IV aGVHD was 24% (95% CI 12–37%) vs. 25% (95% CI 12–41%), respectively. The median survival time for GRFS was 0.3 years (95% CI 0.2–0.5) in both groups. Table 3 describes the survival and GVHD data in further details. In multivariable analysis, PB stem cell source was associated with a decreased likelihood of relapse (HR = 0.17, 95% CI 0.05–0.56, *p* = 0.004), after adjusting for remission status at transplant and area of residence.

## 4. Discussion

In the last two decades, haplo-HCT has undergone revolutionary growth due to its potential to provide a large donor pool to almost everyone who requires an allogeneic stem cell transplant. BMT CTN trial 1101 showed haplo-HCT outperformed cord blood transplant in NRM and OS, with similar PFS, relapse incidence, and GVHD incidence. The trial utilized only BM as a graft source for patients receiving haplo–HCT [13], similar to the original Baltimore protocol developed at John Hopkins University [5]. However, multiple reports have demonstrated that PB is a safe alternative graft source to BM for haplo-HCT graft sources. In a single-center cohort study of 40 consecutive patients between 2012 and 2014, Cieri et al. used melphalan-based myeloablative conditioning and PB haploidentical stem cells and showed an NRM of 17%, PFS of 48%, and OS of 56% at one year. The incidence of grade II-IV aGVHD at day 100 was 15%, and the incidence of cGVHD at one year was 20% [14]. Another phase II trial from Northside Hospital Georgia with 30 patients receiving Flu-TBI conditioning and PB haplo transplant showed NRM of 3%, a relapse rate of 24%, OS of 78%, and disease-free survival of 73% at two years. The incidence of aGVHD (grade II-IV) and cGVHD was 43% and 56%, respectively [15]. Similar outcomes have been reported in other studies for PB stem cell source [9,16].

Our retrospective study compares the outcomes of haplo-HCT between a PB and BM source. Patients in the PB group received more CD3+ and CD34+ cells than the BM, which is consistent with the collection yield of both graft sources [17]. The median time to neutrophil engraftment is similar in BM and PB source (17.5 vs. 16 days, *p* = 0.09), whereas the median time to platelet engraftment is quicker in the PB group (29 vs. 20 days, *p* < 0.001). Bashey et al. showed a similar trend in their analysis with a larger patient population, detecting a significant difference between both groups (BM vs. PB) in count recovery with the median days of 17 vs. 16 (*p* < 0.001) in neutrophil recovery and 26 vs. 25 (*p* < 0.001) days in platelet recovery [11]. While we did not see a similar statistical difference in neutrophil engraftment, this was most likely due to the smaller number of patients included in our cohort. We also had only one incident of graft failure in the PB cohort, which suggests excellent engraftment with a haplo-HCT in our patient population.

Our study shows that BM and PB grafts are similar in outcomes in terms of acute and chronic GVHD, NRM, and GRFS. This is similar to what has been reported previously [18,19,20]. However, a large retrospective CIBMTR database analysis showed a higher incidence of grade II-IV aGVHD and cGVHD in PB graft. This finding may be due to the heterogeneity in groups with a higher number of patients receiving myeloablative doses of TBI (31% in patients in PB group in the Bashey et al. study) [11]. Our data also shows there is a non-significant trend towards improved PFS and OS (49% vs. 68% and 63% vs. 78% at 3 years, respectively), favoring PB.

Our study also demonstrates that the incidence of relapsed disease is significantly lower in PB graft than in BM graft (26% vs. 9% at 1 year). This effect is preserved on multivariable analysis after adjusting for remission status at transplant and area of residence (HR = 0.17, 95% CI 0.05–0.56, *p* = 0.004; Table 4 and Table 5). These results are similar to the ones reported by Bashey et al. in the large retrospective review and O’Donnell et al. [11,18]. The higher dose of CD3+ from donor graft in PB may result in a higher graft-vs-tumor effect. The use of PTCy does not seem to increase the relapse rate beyond that which is expected in a MUD transplant [21,22] and the current BMT CTN 1703 trial compares PTCy to the standard of care in non-myeloablative conditioning regimens in MUD and MRD transplants using PB stem cell source (NCT03959241).

We are aware of limitations of our study. The retrospective design could introduce selection bias into our patient cohort. Moreover, we present a small sample size that limits the study’s power, and subtle differences in outcomes could be missed. This may explain why non-significant OS and PFS are seen in the PB group compared to the BM group. Given that there currently is no published prospective study comparing BM versus PB haplo-HCT, retrospective analyses like ours have helped to guide the use of PB stem cell source.

## 5. Conclusions

Our study suggests that PB is an excellent alternative stem cell source to BM for haplo-HCT. While there was no difference in PFS, OS, GRFS, and NRM, a reduced relapse risk was observed with a PB graft. This is a small retrospective study, but provides encouraging results. A prospective randomized controlled trial is required to confirm these findings.

## Figures and Tables

**Table 1:** Patient Characteristics.

	All (*n* = 74)		BM (*n* = 42)		PB (*n* = 32)		
	N	%	N	%	N	%	*p*-Value
Age at HCT, median, range							0.45
	57.0	20–74	60.0	21–71	54.0	20–74	
Donor age, median, range							0.28
	31.0	19–64	34.0	19–63	28.0	20–64	
Gender, patients							0.67
Male	46	62.2	27	64.3	19	59.4	
Gender, donor							0.13
Male	55	74.3	34	81.0	21	65.6	
Recipient-donor gender							0.10
M–M	35	47.3	24	57.1	11	34.4	
M–F	11	14.9	3	7.1	8	25.0	
F–M	20	27.0	10	23.8	10	31.3	
F–F	8	10.8	5	11.9	3	9.4	
Diagnosis							0.67
AA	1	1.4	1	2.4	0	0.0	
ALL	13	17.6	10	23.8	3	9.4	
AML	25	33.8	14	33.3	11	34.4	
CML	3	4.1	2	4.8	1	3.1	
CLL	7	9.5	3	7.1	4	12.5	
HD	2	2.7	1	2.4	1	3.1	
NHL	11	14.9	5	11.9	6	18.8	
MDS	8	10.8	5	11.9	3	9.4	
MPD	4	5.4	1	2.4	3	9.4	
KPS							0.18
<90	26	35.1	12	28.6	14	43.8	
≥90	48	64.9	30	71.4	18	56.3	
Donor type							0.43
Matched related	1	1.4	0	0.0	1	3.1	
Mismatch related	73	98.6	42	100.0	31	96.9	
GVHD prophylaxis							0.43
FK combination	73	98.6	42	100.0	31	96.9	
Others	1	1.4	0	0.0	1	3.1	
Conditioning							0.54
MA	16	21.6	8	19.0	8	25.0	
RIC	58	78.4	34	81.0	24	75.0	
Comorbidity index, median, range	2.5	0–8	2	0–6	3	0–8	
0–1	27	36.5	18	42.9	9	28.1	0.48
2–3	21	28.4	12	28.6	9	28.1	
4–5	23	31.1	11	26.2	12	37.5	
5+	3	4.1	1	2.4	2	6.3	
Remission status at transplant							0.83
AP	1	1.5	1	2.6	0	0.0	
Chronic phase	1	1.5	1	2.6	0	0.0	
CR	47	72.3	28	71.8	19	73.1	
R/R	5	7.7	4	10.3	1	3.8	
PR	9	13.8	4	10.3	5	19.2	
NA	2	3.1	1	2.6	1	3.8	
cd34 infused, mean, SD	5.66	2.9	3.90	1.7	7.96	2.6	<0.001
cd3 infused, mean, SD	1.28	1.2	0.38	0.2	2.45	1.0	<0.001
Recipient-donor CMV							0.67
Pos-Pos	20	27.0	9	21.4	11	34.4	
Pos-Neg	20	27.0	12	28.6	8	25.0	
Neg-Pos	13	17.6	8	19.0	5	15.6	
Neg-Neg	21	28.4	13	31.0	8	25.0	
Recipient-donor EBV							0.81
Pos-Pos	65	90.3	37	92.5	28	87.5	
Pos-Neg	4	5.6	2	5.0	2	6.3	
Neg-Pos	2	2.8	1	2.5	1	3.1	
Neg-Neg	1	1.4	0	0.0	1	3.1	

Abbreviations: PB, peripheral blood; BM, bone marrow; HCT, transplantation; SD, standard deviation; AA, aplastic anemia; ALL, acute lymphocytic leukemia; AML, acute myeloid leukemia; MM, multiple myeloma; CML, chronic myelogenous leukemia; CLL, chronic lymphocytic leukemia; HD, Hodgkin’s disease; NHL, non-Hodgkins lymphoma; MDS, myelodysplastic syndrome; MPD, myeloproliferative disorder; KPS, Karnofsky score;MA, myeloablative; RIC, reduced intensity conditioning; AP, accelerated phase; CR, complete response; R/R, Relapsed/Refractory; PR, partial response; Pos, positive; neg, negative; GVHD, graft versus host disease; CMV, cytomegalovirus; EBV, Epstein–Barr virus.

**Table 2:** Transplant outcomes of haploidentical donor with BM and PB.

	All (*n* = 74)		BM (*n* = 42)		PB (*n* = 32)		
	N	%	N	%	N	%	*p*-Value
ANC engraftment days, median, range	17	8–31	17.5	8–31	16	13–31	0.09
Platelet engraftment days, median, range	27	13–159	29	19–82	20	13–159	<0.001
Post-transplant response							0.18
CR	66	89.2	35	83.3	31	96.9	
Less than CR	6	8.1	5	11.9	1	3.1	
Progression	2	2.7	2	4.8	0	0.0	
Pulmonary infection							0.82
No	61	82.4	35	83.3	26	81.3	
Yes	13	17.6	7	16.7	6	18.8	
VOD							NA
No	74	100.0	42	100.0	32	100.0	
Bacteremia in first D+100							0.05
No	46	70.8	23	60.5	23	85.2	
Yes	19	29.2	15	39.5	4	14.8	
Viremia in first D+100							0.68
No	25	37.3	15	39.5	10	34.5	
Yes	42	62.7	23	60.5	19	65.5	
Fungemia in first D+100							0.99
No	60	95.2	34	94.4	26	96.3	
Yes	3	4.8	2	5.6	1	3.7	
Hemorrhagic cystitis							0.99
No	65	87.8	37	88.1	28	87.5	
Yes	9	12.2	5	11.9	4	12.5	
Graft failure	1	1.2	0	0.0	1	3.1	0.43
CMV reactivation							
No	38	51.35	22	52.38	16	50	
Yes	36	48.75	20	47.62	16	50	

Abbreviations: CR, complete response; VOD, veno-occlusive disease; D+, Day+; CMV, cytomegalovirus.

**Table 3:** Clinical outcomes, overall and stratified by BM and PB.

	Overall			BM			PB			*p* Value
	Rate	95% CI		Rate	95% CI		Rate	95% CI		
OS										0.13
Median OS, years	NR	3.5	NR	4.3	2.0	NR	NR	NR	NR	
Year 1	76%	64%	84%	71%	55%	83%	81%	63%	91%	
Year 3	69%	57%	79%	63%	46%	76%	78%	59%	89%	
PFS										
Median PFS, years	5.8	1.4	NR	2.2	0.6	NR	NR	2.0	NR	
Year 1	68%	56%	77%	62%	46%	75%	75%	56%	87%	
Year 3	57%	45%	68%	49%	33%	63%	68%	49%	82%	
GRFS										0.9
Median GRFS, years	0.4	0.3	0.5	0.3	0.2	0.5	0.3	0.2	0.5	
Year 1	24%	15%	35%	23%	12%	37%	25%	12%	41%	
Year 3	14%	7%	23%	14%	5%	26%	16%	6%	30%	
NRM										0.76
Year 1	14%	7%	22%	12%	4%	24%	16%	6%	30%	
Year 3	14%	7%	22%	12%	4%	24%	16%	6%	30%	
Relapse										0.03
Year 1	19%	11%	29%	26%	14%	40%	9%	2%	22%	
Year 2	27%	18%	38%	36%	22%	50%	16%	6%	31%	
aGVHD,2–4										
Day 100	59%	47%	70%	57%	41%	70%	63%	44%	77%	0.31
Day 180	59%	47%	60%	57%	41%	70%	63%	44%	77%	
aGVHD, 3–4										
Day 100	24%	15%	35%	24%	12%	37%	25%	12%	41%	0.79
Day 180	24%	15%	35%	24%	12%	37%	25%	12%	41%	
cGVHD, Extensive/Limited										0.18
Day 365	47%	36%	58%	40%	26%	55%	56%	38%	71%	
cGVHD, Extensive										0.97
Day 365	38%	27%	49%	38%	24%	52%	38%	21%	54%	

Abbreviations: PB, peripheral blood; BM, bone marrow; CI, confidence interval; OS, overall survival; PFS, progression free survival; GVHD, graft-versus host disease; a, acute; c, chronic; GRFS, GVHD free relapse free survival; NRM, non-relapse mortality.

**Table 4:** Univariable analysis of effect of PB versus BM graft on transplant outcomes.

	HR	95% CI		*p*-Value
Impact on OS PB vs. BM				
	0.50	0.21	1.21	0.125
Impact on GRFS				
PB vs. BM	1.03	0.63	1.70	0.902
Impact on NRM				
PB vs. BM	1.21	0.38	3.89	0.751
Impact on relapse				
PB vs. BM	0.33	0.13	0.88	0.027

Abbreviations: PB, peripheral blood; BM, bone marrow.

**Table 5:** Multivariable analysis on risk of relapse, adjusting for confounding variables.

	HR	95% CI		*p*
PB vs. BM	0.17	0.05	0.56	0.004
Rural	3.75	1.39	10.08	0.009
Remission status at transplant: CR vs. all others	1.17	0.40	3.43	0.774

Abbreviations: PB, peripheral blood; BM, bone marrow; CR, complete response; HR, hazard ratio.

## Data Availability

This is Institutional data and raw data is not publicly available.
